# Eczéma de contact après tatouage au henné noir

**DOI:** 10.11604/pamj.2013.14.154.2596

**Published:** 2013-04-20

**Authors:** Hayat Bourra, Badreddine Hassam

**Affiliations:** 1 Service de Dermatologie, CHU Ibn Sina, Université Med V, Souissi, Rabat, Maroc

**Keywords:** Eczéma de contact, henné noir, tatouage, Contact Eczema, black Henna, tattoo

## Image en médicine

Le henné ou Lawsonia inermis dit «feuille du paradis» est connu depuis l'antiquité pour ses vertus astringentes, antiseptiques et cicatrisantes, utilisé aussi pour la coloration des cheveux, des mains et des pieds des femmes orientales, et pour la pratique de tatouages labiles en occident. Indolore, peu coûteux, de durée éphémère, il n'expose pas au risque de pathologies transmissibles par le sang. Afin de renforcer la teinte et d'améliorer la fixation du henné, on rajoute des agents colorants soit la paraphénylène diamine (PPD) ou le diaminotoluène qui peuvent être responsables d'eczémas de contact, parfois des sensibilisations croisées avec d'autres substances; la réaction allergique au henné pur est très rare. Indépendamment du pouvoir sensibilisant propre à la PPD, le risque de sensibilisation est d'autant plus grand que la concentration moléculaire et la durée du contact avec la molécule sont importantes. Le taux de concentration de la PPD inférieur à 6 %, autorisé par l'Union européenne, est respecté pour la plupart des teintures capillaires et des colorants textiles. En revanche, la concentration de PPD lors de tatouages au henné noir reste bien moins certaine, surtout lorsque l'on sait que ces tatouages sont préparés de façon artisanale ne se soumettant à aucune norme de fabrication ni de dosage quantifié. Nous rapportons l'observation d'une patiente de 15 ans qui a présenté 48 h après un tatouage au henné noir des lésions érythémato-vésiculeuses et œdémateuses très prurigineuses au site du tatouage reproduisant le dessin initial, traitée par un dermocorticoïde de classe forte une application par jour pendant une semaine, puis un jour sur deux pendant une autre semaine avec régression de l'eczéma mais persistance de lésions hypopigmentées.

**Figure 1:**
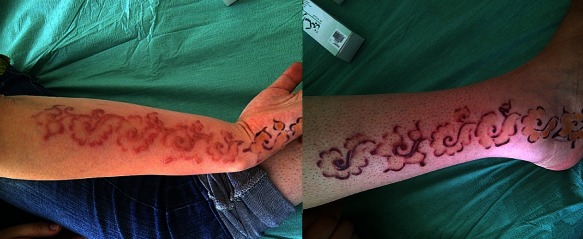
Lésions vésiculo-bulles avant-bras gauche, lésions œdémateuses de la jambe droite

